# Role of Noncoding RNAs in Modulating Microglial Phenotype

**DOI:** 10.1055/s-0044-1790283

**Published:** 2024-09-09

**Authors:** Eiman Meer

**Affiliations:** 1 Department of Biological and Health Sciences, Pak-Austria Fachhochschule Institute of Applied Sciences and Technology, Haripur, Pakistan

**Keywords:** noncoding RNAs, microRNA, long noncoding RNAs, microglia, inflammation

## Abstract

Microglia are immunocompetent cells that are present in the retina and central nervous system, and are involved in the development maintenance and immune functions in these systems. Developing from yolk sac-primitive macrophages, they proliferate in the local tissues during the embryonic period without resorting to the production from the hematopoietic stem cells, and are critical in sustaining homeostasis and performing in disease and injury; they have morphological characteristics and distinct phenotypes according to the microenvironment. Microglia are also present in close association with resident cells in the retina where they engage in synapse formation, support normal functions, as well as immune defense. They are involved in the development of numerous neurodegenerative and ophthalmic diseases and act as diversity shields and triggers. Noncoding ribonucleic acids (ncRNAs) refer to RNA molecules synthesized from the mammalian genome, and these do not have protein-coding capacity. These ncRNAs play a role in the regulation of gene expression patterns. ncRNAs have only been recently identified as vastly significant molecules that are involved in the posttranscriptional regulation. Microglia are crucial for brain health and functions and current studies have focused on the effects caused by ncRNA on microglial types. Thus, the aim of the review was to provide an overview of the current knowledge about the regulation of microglial phenotypes by ncRNAs.

## Introduction

### Background

#### Microglia

Microglia are inhabitant immune cells in the central nervous system (CNS) and in retina of the eye. They play key roles in the development and normal functioning of immune response in these systems. 1 They originate from sac-primitive macrophages and flourish in situ during the embryonic period. 2 Once developed, the microglial population develops and proliferates locally, without support from hematopoietic progenitor cells. 3

Microglia are the key players of nervous immune system by playing a pivotal role in maintaining homeostasis and responding to any disease and injury. They show morphological changes in response to changes in their environment, and switch between distinct phenotypes. On the basis of their phenotypes, these cells are classified as proinflammatory and anti-inflammatory phenotype. 4 Microglial response to tissue damage is mediated by neurotransmitters and receptors that get activated in the presence of injury-related molecules such as pathogen-associated molecular patterns (PAMPs), 5 danger-associated molecular patterns (DAMPs), 6 lipopolysaccharides, 7 and pathogen-derived nucleic acids. 8

**Fig. 1:**
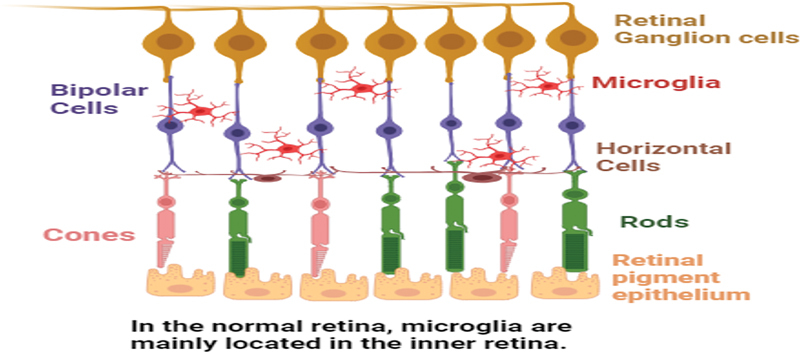
Depiction of normal retina.

**Fig. 2:**
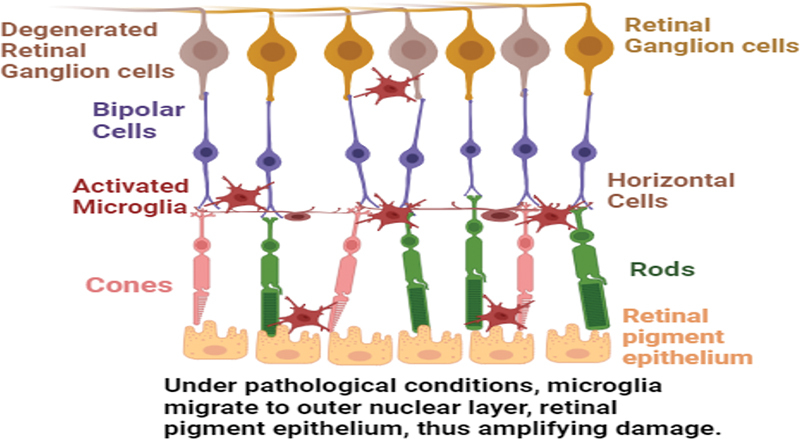
Activated microglia causing retinal damage.

In the retina, microglia exist as an integral component of the retinal cells population, and they resemble microglial cells in the CNS in morphological aspects when are in resting state and even during the activate form. 9 Depicted in Fig. 1 , under normal physiological conditions in the eye, microglial cells of retina reside in inner retina, while when they are activated, they move toward outer retina. 10 As depicted in Figs. 1 and 2 , respectively.

In retinal development, microglial cells have indirect physical contacts with other glial cells and neurons for the forming of synapses, regulating the homeostasis and performing immune functions. When engaged, the microglia play a role in pathogenesis, progression, and outcome of multiple neurodegenerative and ophthalmic diseases. 11 Microglia are supposed to be a two-edged weapon in the retina, they could initiate inflammation response and proapoptosis besides pondering its protection. 12

The ability of microglia to exist in proinflammatory (M1) and anti-inflammatory (M2) phenotypes is crucial for their function, and research has highlighted upon the function of noncoding ribonucleic acids (ncRNAs) in regulating microglial phenotype. 13 The objective of this review was to gather the available information about the role of ncRNAs in modulating microglial phenotype and their role as potential therapeutic targets.

#### Distinct Features of Proinflammatory and Anti-Inflammatory Microglia Phenotypes

Activated microglia upregulate their surface proteins like CD86 and MHC II that help in antigen presentation and turning on T cells. 14 15 Also, they generate proinflammatory cytokines such as interleukin (IL)-6 and tumor necrosis factor-alpha (TNF-α) but decreased levels of IL-10. In contrast, the anti-inflammatory microglia bear antigens that include CD206 and release factors that are linked with immunosuppressive characteristics and tissue repair such as transforming growth factor (TGF)-β and IL-10. 4 16 This switch entails activation of genes involved in immune response and inflammation including, IL-1β, TNF-α, and inducible nitric oxide synthase. Specific genes are executed through various transcription factors such as nuclear factor kappa B (NF-κB) and signal transducer and activator of transcription 1 (STAT1). 17 18 Contrary to this, anti-inflammatory state of microglia increase the expression of genes included in the Arg1, Ym1, and IL-10, regulated by transcriptional factors like STAT6 and peroxisome proliferator-activated receptor gamma (PPARγ), which are responsible for terminating tissue repair and inflammation. 19 20

In the CNS, the microglia become proinflammatory, acquiring an amoeboid form accompanied by shrinkage of the processes and enlargement of the cell body. This morphology suits their pharmacokinetic profile and the fact they are generally in an active, phagocytic state and are more motile than their sedentary counterparts. 21 22 On the other hand, anti-inflammatory microglia have a ramified shape with numerous, long slender processes compatible with monitoring the tissue environment and keeping the balance. 23 24

Activated microglia show high densities of proteins that are engaged in antigen presentation from the external environment and initiation of adaptive immune responses, these include proteins CD86 and MHC class II. 14 15 Also, they secrete proinflammatory cytokine on the pattern of TNF-α and IL-6 indices into the blood. M2 microglia, at the same time, presents specific markers, for instance, CD206 and releasing anti-inflammatory mediators encompassing TGF-β and IL-10 that lead to a reparative response and immunosuppression. 4 16

This is also true for receptors because there are changes in their expression between the phenotypes. Activated microglial receptor leads to the activation of microglia and upregulation of Toll-like receptors (TLRs), especially TLR4 which has the capacity to identify PAMPs and DAMPs. M2 microglia have higher receptor density for such anti-inflammatory cytokines as IL-10R and IL-4R and they are bound to shift the microglia to M2 state. 1

Subsequently, microglia in the eye including the retina exhibit the similar functional flexibility as observed in the CNS. They are involved in bid essential tasks regarding the homeostatic regulation of retinal tissue as well as its response to injury or disease. That is why differentiation between anti-inflammatory and proinflammatory phenotypes is important for the consideration of the illness course and possibly its alteration. The retinal microglia are also primed to a proinflammatory phenotype, and similar to their CNS derivatives they appear morphologically activated, with a rounded cell body and retracted processes. Anti-inflammatory retinal microglia are less activated characterized by a more branched, ramified shape ideal for their purpose of patrolling and keeping order. 25 The proinflammatory phenotype involves upregulation of genes coding proteins like IL-1β, TNF-α, and CXCL10 regulated by transcription factors STAT1 and NF-κB in the retina. 26 Anti-inflammatory microglia release molecules like Arg1 and IL-10 for tissue repair and play immunosuppressive functions with the help of STAT6 and PPARγ pathways. 27 CD86 and MHC II are markers of physique of proinflammatory microglial cells, and they emerge as IL-6 and TNF-α in the retinal cells that cause inflammation which may drive retinal degenerative diseases. 28 Anti-inflammatory microglia have specific markers including CD206 and release cytokines including TGF-β and IL-10, which help the clearing of inflammation and repair of the retinal tissue. 29

These proinflammatory retinal microglia increase receptors such as TLR4 that participates in recognition and response to pathogens and signals for injury. 23 It also demonstrated that anti-inflammatory microglia has higher IL-4R and IL-10R, which help them to respond to prohealing signals and hence turn into the refreshing phenotype. 18

#### Noncoding RNAs

**Fig. 3:**
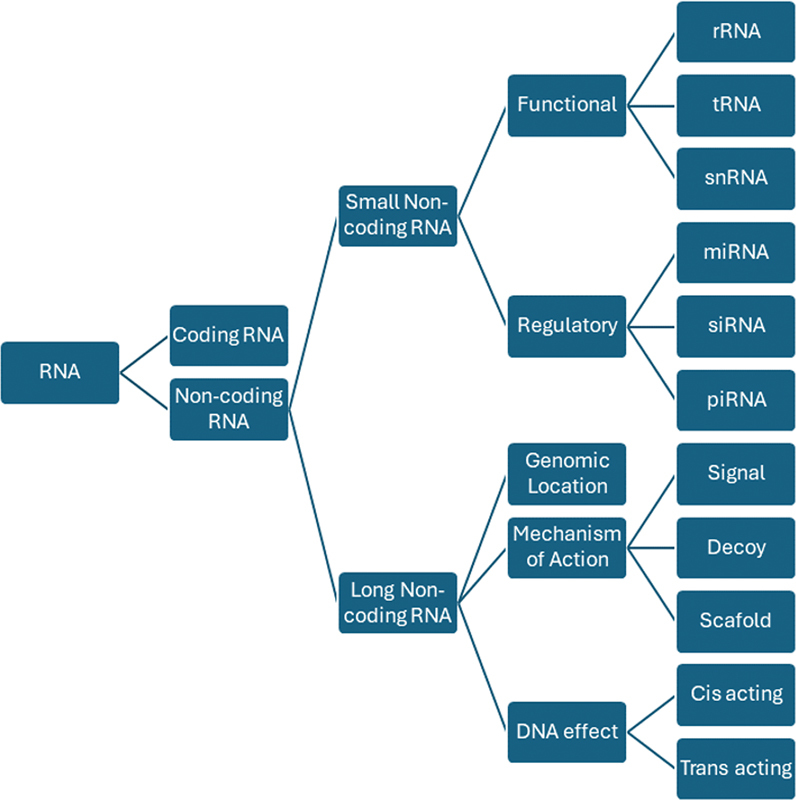
Classes of ribonucleic acid (RNA).

ncRNAs are RNA molecules transcribed from the mammalian genome that do not code for proteins. About 80% of human genome consists of noncoding elements, including small ncRNAs like microRNAs (miRNA), siRNA, piRNA, and snRNA, as well as long ncRNAs (lncRNAs) including long intergenic RNAs (lincRNA), natural antisense transcripts, enhancer RNAs, circular RNAs (circRNAs), competing endogenous RNAs (ceRNAs), and PROMoter uPstream Transcripts (PROMPTS). 30 These ncRNAs play key roles in regulating gene expression. In recent years, ncRNAs have earned recognition as crucial molecules in posttranscriptional regulation, functioning as transcriptional enhancers, guides, scaffolds, and decoys, in addition to their roles in transcription. 30 Fig. 3 provides an overview of the types of ncRNAs and their functional roles.

### Rationale and Knowledge Gap

The ability of microglia to exist in proinflammatory (M1) and anti-inflammatory (M2) phenotypes is crucial for their function, and research has highlighted upon the function of ncRNAs in regulating microglial phenotype. 13 Thus, this review gathers the available information about ncRNAs involved in microglial phenotype determination.

### Objective

The objective of this review is to gather the available information about the role of ncRNAs in modulating microglial phenotype and their role as potential therapeutic targets.

## Main Body

### Role of ncRNAs in Modulating Microglial Phenotype

These are little RNA fragments which have the ability to reproduce their complimentary sequence on designated messenger RNAs (mRNAs), hence leading to destroy or not to translate. They range for approximately between 18 and 22 nucleotides in size. lncRNAs are greater than 200 nucleotides and can modulate gene expression at various levels like at deoxyribonucleic acid (DNA), RNA, and protein level which includes chromatin modification, transcription regulation, and posttranscriptional protein modifications. CircRNAs are a recently described fresh class of ncRNA created by back-splicing exons, work as miRNA sponges, regulating the accessible accessibility of miRNA to its target mRNAs. These ncRNAs have been found to control various cellular processes, including alteration of microglial phenotype.

### Types of Noncoding RNAs Involved in Microglial Phenotype Modulation

#### MicroRNAs

miRNAs are a member of ncRNA family, with profound role in gene regulation, by interacting with target mRNAs. Primary function of miRNAs is to control protein synthesis by mRNA degradation or translational repression, 31 but several studies have demonstrated that some miRNAs also enhance the expression of their target mRNAs in certain cell types (Place et al, 2008). 63 Mature miRNAs are 18 to 22 nucleotide long RNA molecules, that are processed from > 100 base pairs long primary miRNAs (pri-miRNAs) transcripts. 32 Pri-miRNAs are characterized stem-loop structures, 33 that are recognized and cleaved by a nuclear enzyme Drosha, 34 along with the action of an RNA-binding protein (RBP) Pasha/DGCR8 which is a component of almost 650 kDa, microprocessor complex. 35 The processing of pri-miRNAs by microprocessor complex leads to the production of pre-miRNAs, that are 70 nucleotides long RNA molecules, which are transported out of the nucleus by a nuclear receptor named exportin-5, 36 that specifically binds with pre-miRNAs.

miRNAs are the “master regulators” of gene expression by targeting multiple genes across biological pathways by controlling distinct physiological outcomes such as cell proliferation, differentiation, metabolism, apoptosis, immune responses, and stress responses. 37 In additive to their role in various physiological processes, miRNAs also play significant roles in microglial activation and phenotype switching. The stability between proinflammatory (M1) verses anti-inflammatory (M2) microglial states is essential for maintaining CNS homeostasis and responding to pathological conditions. Dysregulation of miRNAs can lead to chronic inflammation and neurodegeneration.

#### miR-124

miR-124 (Gene ID: 406904, Chromosomal Location: 8p23.1) is among the most commonly expressed miRNAs in the CNS and is involved in the development of anti-inflammatory (M2) phenotype of microglia. This anti-inflammatory phenotype is achieved by inhibiting a transcription factor, CCAAT/enhancer-binding protein-α (C/EBP-α), and its downstream target PU1 that are involved in the activation of the proinflammatory M1 state. 38 Upregulation of miR-124 in microglia results in lower production of proinflammatory cytokines such as and IL-1β and TNF-α, thereby reducing neuroinflammation and enhancing neuroprotection. 38 Another study has shown that miR-124 suppresses the expression of repressor element 1-silencing transcription factor (REST), that leads to increased expression of anti-inflammatory genes. 39

#### miR-155

In contrast to miR-124, miR-155 (Gene ID: 406947, Chromosomal Location: 21q21.3) is connected with promoting the M1 proinflammatory phenotype. During neuroinflammatory conditions, miR-155 levels rise in microglia and it acts by binging to suppressor of cytokine signaling 1 (SOCS1), which is a negative regulator of the inflammatory response. 40 By restraining SOCS1, miR-155 boosts the cellular synthesis of proinflammatory cytokines that will drive microglia toward M1 state, thus promoting inflammatory response. Elevated levels of miR-155 have been linked in several neurodegenerative diseases, including multiple sclerosis and Alzheimer's disease (AD), where it intensifies neuroinflammation and thus ultimately damaging the neurons. 40 41

#### miR-146a

miR-146a (Gene ID: 406936, Chromosomal Location: 5q33.3) is involved in the regulation of microglial phenotype, particularly in the context of inflammatory responses. mir-146a acts as negative feedback regulator to control excessive inflammation. miR-146a targets and suppresses expression of two key adapter molecules in NF-κB signaling pathway, named TRAF6 (tumor necrosis factor receptor-associated factor 6) and IRAK1 (interleukin-1 receptor-associated kinase 1). 42 In this manner, miR-146a reduces triggering of NF-κB signaling pathway, and thus reducing production of proinflammatory cytokines. In response to inflammatory stimuli, a higher expression of miR-146a has been observed in microglia, indicating its function in reducing excessive inflammatory responses and promoting transfer toward the M2 phenotype. 42

#### miR-21

Depending on the physiological conditions, miR-21 (Gene ID: 406991, Chromosomal Location: 17q23.1) is correlated to both proinflammatory and anti-inflammatory effects. Main cellular target of miR-21 include programmed cell death protein 4 (PDCD4), that is involved in both apoptosis and inflammation. 43 In case of CNS damage in injuries, miR-21 has been shown to decrease the expression of PDCD4, by hindering apoptosis and promoting cell survival. miR-21 is also observed to increase the phagocytic activity of microglia, and thus it contributes to the clearance of debris and damaged cells. 43 This dual role highlights the complexity of miRNA-mediated regulation of microglial phenotypes and their potential therapeutic applications.

#### miR-223

miR-223 (Gene ID: 407022, Chromosomal Location: Xq12) is another member of class of miRNAs that has a significant role in modulating microglial activation. It has been observed that miR-223, by targeting the transcription factor STAT3, promote anti-inflammatory (M2) phenotype, which is involved in the M1 activation pathway. 44 As an inhibitor of STAT3, miR-223 reduces the expression of proinflammatory cytokines and alternatively, it promotes the expression of anti-inflammatory markers. The upregulation of miR-223 has been observed as a protective effect in mice models of CNS inflammation, proposing its capability as therapeutic target for modulating microglial responses. 44

### Long Noncoding RNAs (lncRNAs)

lncRNAs are a large group of RNA molecules that are generally more than 200 nucleotides in length. These lncRNAs act as essential players in the gene expression to control it in the several levels including the transcriptional level, chromatin level, and posttranscriptional level on the mRNAs. They might also promote lncRNAs as signaling or guide molecules, scaffolds, or decoys and the function of such molecules is performed typically by interacting with their target DNA, RNA, and proteins. 45

lncRNAs are signaling molecules, hence showing transcriptional activity of specific loci. They can be turned on regarding different stimuli, corresponding to cell statuses. They can act as receptors that recruit chromatin-modifying complexes to certain regions in the genome thus altering the epigenetic environment and subsequently gene expression. Some lncRNAs act as a shell to construct several protein complexes to accommodate interacting of proteins and nucleic acids. lncRNAs can also be regulatory RNA and perform the function of sinks to trap transcription factors, RNA-associate proteins, or miRNAs to influence the activity of the encoded proteins and thus the expression of directly targeted genes. 46

Thus, performing their biological roles through these mechanisms, lncRNAs are implicated in controlling numerous developmental, differentiation, and immune processes. In the CNS settings, lncRNAs have a powerful impact on microglial activation and PD-like phenotype transition, as well as activation involving neuroinflammatory and neurodegenerative processes.

### lncRNAs Involved in Microglial Phenotype Regulation

#### lncRNA-CCL2

lncRNA-CCL2 (Gene ID: 6347, Chromosomal Location: 17q12) is a lncRNA and has a central function in microglial activation. It modulates the production of CCL2 or monocyte chemoattractant protein-1, which plays a crucial role in microglial activation and attraction of other immune cells into the CNS. Some investigations have shown that lncRNA-CCL2 is increased in the microglia under inflammatory conditions including lipopolysaccharide (LPS) and amyloid-β. 47 Thus, lncRNA-CCL2-mediated regulation of CCL2 accounts for the proinflammatory state of microglia involved in neuroinflammation and neurodegeneration in diseases such as AD.

#### lncRNA-MEG3

There are other lncRNAs (Gene ID: 55384, Chromosomal Location: 14q32.2) that are involved in the process of microglial activation, one that could be mentioned is lncRNA MEG3 (maternally expressed gene 3). Expressed in many different types of cancer, MEG3 is a tumor suppressor, despite the fact that it has imperative roles in CNS. MEG3 can also control inflammatory-related genes via ceRNA to miRNA and in which MEG3 can bind to miR-21 and relieve the target gene STAT3. 48 Thus, MEG3 can indeed reduce the impact of LPS increase in miR-21 and thereby also counter the effect of STAT3 in the inflammation of microglia. In accomplishing this, it regulates the flip between the nasty M1 macrophages and the fixer M2 macrophages to the loss of neuronal tissue and inflammation decrease.

#### LncRNA HOTAIR

LncRNA HOTAIR (Gene ID: 100124700, Chromosomal Location: 2q13.13) was earlier reported to be a modulator of inflammation through participation of microglial activation via the NF-κB signaling pathway. 49

#### lncRNA-MALAT1

MALAT1 (Gene ID: 378938, Chromosomal Location: 11q13.1) is one such lncRNA gene that has a crucial function in deciding the microglial response. All tissues exhibit MALAT1 expression, and they reported that it covers all aspects of biological processes ranging from the cell division, mobility, to immunological responses of the CNS. 44 When it comes to inflammation regulation, the NF-κB nuclear factor signaling is held to be impacted by the microglial MALAT1. A study has also described how MALAT1 can induce activation of NF-κB that results in increasing the production of IL-6 and TNF-α, as a contribution to M1 macrophage phenotype. 50

#### lncRNA-Sox2OT

Sox2 overlapping transcript (Sox2OT) (Gene ID: 347689, Chromosomal Location: 3q26.33) is a lncRNA and is located adjacent to the Sox2 gene which is important to the stem cell potency as has been pointed. Last but not the least, Sox2OT is involved in the developmental and differentiative processes of a multimeric organism for which it is a master regulator of genes among other things.

With regards to microglial regulation, it is worth noting that Sox2OT has been revealed as interacting with the transcription factor Sox2 and alters its activity, thus affecting the activation of microglia. 51 Thus, neurotrophic Sox2OT activity increases after LPS treatment and stimulates the production of anti-inflammatory cytokines, including IL-10, which leads to the M2 phenotype. This regulation aids in regulating inflammation and protects the neurons during CNS injuries and in neurodegeneration. Table 1 Enlists different types of ncRNAs and their role in microglial phenotype determination.

**Table 1:** Enlisting different noncoding RNAs and their role in microglial phenotype

Noncoding RNA	Target molecule	Role in microglial phenotype determination	Reference
**miR-124**	PU.1	Promotes anti-inflammatory phenotype	38
**miR-155**	SOCS1	Promotes proinflammatory phenotype	40
**miR-146a**	IRAK1, TRAF6	Regulates inflammation and immune response	42
**miR-21**	PTEN	Modulates inflammation and promotes survival	58
**miR-223**	NF-κB	Reduces proinflammatory cytokine production	59
**lncRNA-MEG3**	NF-κB	Inhibits inflammation and apoptosis	60
**lncRNA-MALAT1**	STAT1	Modulates inflammation and apoptosis	61
**lncRNA-SNHG14**	Bcl-2	Regulates cell proliferation and apoptosis	62

Abbreviations: IRAK1, interleukin-1 receptor-associated kinase 1; NF-κB, nuclear factor kappa B; PTEN, phosphatase and tensin homolog; RNA, ribonucleic acid; SOCS1, suppressor of cytokine signaling 1; STAT1, signal transducer and activator of transcription 1; TRAF6, tumor necrosis factor receptor-associated factor 6.

### Circular RNAs

circRNAs are new kind of ncRNA circles, which are generated through back-splicing events, during mRNA splicing, and formation of a covalently closed loop structure. Such arrangement makes circRNAs more stable than linear RNAs because they are not capped at the 5 ends and polyadenylated at the 3 ends, thus cannot be degraded by exonucleases. 52

One of the main functions that have been associated with circRNAs is that they can act as miRNA sponges which sequester miRNAs and do not allow them to downregulate their target mRNAs. This type of activity depends on whether or not it sponges miRNA and as a result can indirectly affect the corresponding genes. 53 Moreover, circRNAs could bind to RBPs to promote or block the assembly of RBP complexes, and rarely as short open reading frame-containing circRNAs, they can encode for peptides. 54 55

### circRNAs Involved in Microglial Phenotype Modulation

Increased importance of circRNAs in the regulation of microglial functions and phenotype has been reported. Thus, circHIPK3 is one of the identified factors that can regulate inflammation. CircHIPK3 (Gene ID: 28996, Chromosomal Location: 11p13) binds directly to miR-124, and as mentioned earlier, miR-124 is a miRNA which has been proved to have anti-inflammatory effect. CircHIPK3 may modulate the expression of the transcription factor STAT3, which is implicated in the amplification of an inflammatory response in microglia, through sponging miR-124. 56 It has been identified that circSLC8A1 (Gene ID: 6546, Chromosomal Location: 2p22.1) affects microglial apoptosis and inflammation through absorbing miR-128–3p. The above interaction results in the increased expression of Bcl-2 proteins that helps in preventing apoptosis and there is also the suppression of the content of proinflammatory cytokines. These findings point toward circSLC8A1's function in responding to inflammation and maintaining microglial homeostasis, which implies circSLC8A1-related therapeutic insights on neuroinflammatory diseases. 57
